# Characterization of Root System Architecture Traits in Diverse Soybean Genotypes Using a Semi-Hydroponic System

**DOI:** 10.3390/plants10122781

**Published:** 2021-12-16

**Authors:** Shuo Liu, Naheeda Begum, Tingting An, Tuanjie Zhao, Bingcheng Xu, Suiqi Zhang, Xiping Deng, Hon-Ming Lam, Henry T. Nguyen, Kadambot H. M. Siddique, Yinglong Chen

**Affiliations:** 1 College of Natural Resources and Environment, and State Key Laboratory of Soil Erosion and Dryland Farming on the Loess Plateau, Northwest A&F University, Xi’an 712100, China; liushuo@nwafu.edu.cn (S.L.); tingtingan2018@nwafu.edu.cn (T.A.); bcxu@ms.iswc.ac.cn (B.X.); sqzhang@ms.iswc.ac.cn (S.Z.); dengxp@ms.iswc.ac.cn (X.D.); 2 National Center for Soybean Improvement, Key Laboratory of Biology and Genetics and Breeding for Soybean, Ministry of Agriculture, State Key Laboratory for Crop Genetics and Germplasm Enhancement, Nanjing Agricultural University, Nanjing 210095, China; T2020106@njau.edu.cn (N.B.); tjzhao@njau.edu.cn (T.Z.); 3 Center for Soybean Research of the State Key Laboratory of Agrobiotechnology, School of Life Sciences, The Chinese University of Hong Kong, Shatin, Hong Kong, China; honming@cuhk.edu.hk; 4 Division of Plant Sciences, University of Missouri, Columbia, MO 65211, USA; NguyenHenry@missouri.edu; 5 The UWA Institute of Agriculture, School of Agriculture and Environment, The University of Western Australia, Perth 6009, Australia; kadambot.siddique@uwa.edu.au

**Keywords:** soybean, root phenomics, root system architecture, root distribution

## Abstract

Phenotypic variation and correlations among root traits form the basis for selecting and breeding soybean varieties with efficient access to water and nutrients and better adaptation to abiotic stresses. Therefore, it is important to develop a simple and consistent system to study root traits in soybean. In this study, we adopted the semi-hydroponic system to investigate the variability in root morphological traits of 171 soybean genotypes popularized in the Yangtze and Huaihe River regions, eastern China. Highly diverse phenotypes were observed: shoot height (18.7–86.7 cm per plant with a median of 52.3 cm); total root length (208–1663 cm per plant with a median of 885 cm); and root mass (dry weight) (19.4–251 mg per plant with a median of 124 mg). Both total root length and root mass exhibited significant positive correlation with shoot mass (*p* ≤ 0.05), indicating their relationship with plant growth and adaptation strategies. The nine selected traits contributed to one of the two principal components (eigenvalues > 1), accounting for 78.9% of the total genotypic variation. Agglomerative hierarchical clustering analysis separated the 171 genotypes into five major groups based on these root traits. Three selected genotypes with contrasting root systems were validated in soil-filled rhizoboxes (1.5 m deep) until maturity. Consistent ranking of the genotypes in some important root traits at various growth stages between the two experiments indicates the reliability of the semi-hydroponic system in phenotyping root trait variability at the early growth stage in soybean germplasms.

## 1. Introduction

Soybean (*Glycine max* L. Merr.) is an important leguminous crop that is used for human food, animal feed, biofuel production, and many other products due to its high protein and edible oil content [1]. Soybean is native to China and is the fourth largest crop after rice (*Oryza sativa* L.), wheat (*Triticum aestivum* L.), and corn (*Zea mays* L.) of the world. It is grown on an estimated 6% of the world’s arable land [1,2]. In China, the region between the Yangtze and Huaihe Rivers is among the major soybean-producing area [2]. Studies have investigated crop growth and yield constraints, and there is increasing interest in soybean varieties with suitable root systems to effectively use resources and improve yield [3,4,5]. Plant root systems play an essential role in water and nutrient acquisition, and they can perceive and respond to various edaphic stresses before other plant organs, such as drought, waterlogging, salinity, and soil infertility [6,7,8,9,10,11,12,13].

Root system architecture (RSA) is plastic and dynamic, allowing plants to respond to their environments to enhance nutrients and water acquisition [14,15]. Alterations to root growth and RSA are critical adaptive strategies of crops to cope with drought, soil infertility, and other edaphic stresses [16]. Therefore, it is important to screen and breed crop cultivars with better RSA, which can adapt to edaphic stresses and have improved nutrient and water efficiency [17,18]. The identification of RSA conferring efficiencies in resource acquisition and adaptation to edaphic stresses has increased in research and breeding programs [19,20]. Some studies have evaluated the phenology and morphological characters of many soybean genetic resources [6,21]. Research on soybean in the Yangtze and Huaihe River region of eastern China has mainly focused on seed traits, pod traits, shoot traits, and flowering time [22,23,24,25,26,27,28]. Studies on soybean root traits are limited due to the lack of reliable and efficient non-destructive methods [29,30].

A semi-hydroponic phenotyping system [31] has been used to characterize root traits in large numbers of germplasm in various crop species, including narrow-leafed lupin (*Lupinus angustifolius* L.) [32,33,34], chickpea (*Cicer arietinum* L.) [35], maize (*Zea mays* L.) [36], wheat (*Triticum aestivum* L.) [37,38], and barley (*Hordeum vulgare* L.) [39], but not in soybean. In this study, we characterized phenotypic variability in root morphology at the seedling stage of the 171 diverse genotypes with good agronomic traits mainly developed for the Yangtze and Huaihe Rivers in eastern China. The root growth of three selected soybean genotypes was mapped in a follow-up validation study in soil-filled deep rhizoboxes.

## 2. Results

### 2.1. Adopting a Semi-Hydroponic System to Characterize 171 Soybean Accessions

#### 2.1.1. Global Traits

Global traits include nine root traits and four shoot traits (Table 1). The nine root traits reflected the pattern of root distribution and development. Among them, maximal root depth (CV = 0.16), root width (CV = 0.18), root angle (CV = 0.26), total root length (CV = 0.37), average root diameter (CV = 0.07), root mass (CV = 0.44), and root–shoot mass ratio (CV = 0.27) significantly different among genotypes (all *p* < 0.01), while the specific root length (CV = 0.49) and root tissue density (CV = 0.28) did not significantly differ among genotypes (Table 2). Four shoot-related traits, leaf number (CV = 0.14), hypocotyl length (CV = 0.27), shoot height (CV = 0.22), and shoot mass (CV = 0.33) significantly differed among genotypes (all *p* < 0.01; Table 2).

**Table 1:** Description of 18 measured traits (13 global and five local traits) in 171 soybean genotypes grown in a semi-hydroponic phenotyping platform assessed at 39 days after sowing.

Traits	Abbreviation	Description	Units
Global traits (traits at the whole plant level)			
Maximal root depth	MRD	Longest seminal root length, i.e., rooting depth	cm
Root width	RW	Maximal horizontal extent of a root system (Figure 1c)	cm
Root angle	RA	Growth angle between two outer lateral roots (Figure 1c)	Degree
Total root length	RL	Total root length per plant	cm
Root diameter	RD	Average diameter of root system per plant	mm
Root mass	RM	Total dry mass of roots per plant	mg
Specific root length	SRL	Total root length divided by root dry mass	cm mg^−1^
Root tissue density	RTD	Total root dry mass divided by root volume	mg cm^−3^
Root–shoot mass ratio	RSM	Total root dry mass divided by shoot dry mass	
Leaf number	LN	Number of leaves per plant	
Hypocotyl length	HL	Length from cotyledon node to the origin on the primary root	cm
Shoot height	SH	Shoot height (measured at its height from cotyledon node to plant growth point)	cm
Shoot mass	SM	Total shoot dry mass per plant	mg
Local traits (traits at local level including ratios)			
Root length—upper	RL-upper	Root length in upper 0–20 cm soil layer	cm
Root length—lower	RL-lower	Root length below 20 cm depth	cm
Root length in diameter fine	RL-fine	Root length of ‘fine roots’ (in diameter class < 0.5 mm)	cm
Root length in diameter coarse	RL-coarse	Root length of ‘coarse roots’ (diameter class ≥ 0.5 mm)	cm
Root length ratio	RLR-upper/lower	Root length in top 20 cm soil layer divided by in root length below 20 cm soil depth	cm

The same set of traits (except maximal root depth, root width, root angle, and leaf number) were measured in three soybean genotypes (#054, #055, and #071) grown in soil-filled rhizoboxes assessed at 38 and 81 days after sowing. Root mass, root–shoot mass ratio, hypocotyl length, shoot height, and shoot mass were assessed 160 days after sowing.

Root mass ranged from 19.4 (genotype #071) to 251 (#055) mg per plant with a median value of 124 mg (Table 2). About 63% of the 171 genotypes had root mass values > 110 mg per plant. Total root length (RL) ranged from 208 (#071) to 1663 (#055) cm per plant with a median value of 885 cm (Table 2, Figure 2). Among the 171 genotypes, four genotypes had RL < 300 cm per plant, 157 genotypes had RL values from 300 to 1300 cm per plant, and ten genotypes had RL > 1300 cm (Figure 2). Based on the RL value of each genotype compared to the median value (885 cm per plant) of RL ± two standard error, the 171 soybean genotypes were divided into three root sizes; 58 genotypes were considered as having a small root system (RL < 850 cm per plant), 68 genotypes were considered as having a large root system (RL > 913 cm per plant), and 45 genotypes were considered as having a medium root system (850 ≤ RL ≤ 913 cm per plant) (Table S1). The root mass, root length distribution of fine roots (diameter class < 0.5 mm), and coarse roots (diameter class ≥ 0.5 mm) followed similar trends to RL for the three root size classes (Figure 3).

Among the 68 genotypes from the northern Huaihe River region (NHHR), 29 genotypes had large root systems, 23 genotypes had medium root systems, and 16 genotypes had small root systems (Table S1). Among the 75 genotypes from the southern Huaihe River region (SHHR), 30 genotypes had large root systems, 17 genotypes had medium root systems, and 28 genotypes had small root systems (Table S1). Among the 28 parental lines (PL), 9 genotypes had large root systems, 5 genotypes had medium root systems, and 14 genotypes had small root systems (Table S1). The differences between the maximum and the minimum values of root mass, total root length, root length distribution of fine roots (diameter class < 0.5 mm), and coarse roots (diameter class ≥ 0.5 mm) were more profound than those of NHHR and SHHR genotypes, respectively (Figure S1).

Variation also occurred for maximal rooting depth, root width, root angle, average root diameter, and root–shoot mass ratio. Genotype #001 had the greatest maximal rooting depth (65 cm) per plant, which was 2.3-fold deeper than the shortest rooting depth (28.8 cm, #079). Root width ranged from 9.57 (#002) to 20.5 (#120) cm per plant. Root angle ranged from 63° (#171) to 160° (#068), with about 84% of the 171 genotypes > 90°. Average root diameter ranged from 0.20 (#079) to 0.47 (#077) mm per plant, and the root–shoot mass ratio ranged from 0.11 (#168) to 0.32 (#034) (Table 2).

At harvest (39 DAS), six genotypes had three trifoliate leaves, and five genotypes had six trifoliate leaves. The longest hypocotyl length was 9.1 (#131) cm per plant, and the shortest hypocotyl length was 2.2 (#014) cm per plant. Shoot height ranged from 18.7 (#071) to 86.7 (#023) cm per plant, with about 56% of the 171 genotypes > 51 cm. Shoot mass ranged from 164 (#071) to 1150 (#023) mg per plant (Table 2).

#### 2.1.2. Local Traits

Root traits at different depths and root lengths in different diameter classes (Table 1 and Table 2) varied significantly with CVs ≥ 0.3. The root length (upper), root length in the upper 0–20 cm soil layer, ranged from 152 (genotype #071) to 1146 (#030) cm per plant (CV = 0.34). Root length (lower), root length below 20 cm soil depth, ranged from 11.5 (#079) to 654 (#001) cm per plant (CV = 0.58). Root length (fine), diameter class < 0.5 mm, ranged from 134 (#079) to 908 (#165) cm per plant (CV = 0.46). Root length (coarse), diameter class ≥ 0.5 mm, ranged from 175 (#071) to 1551 (#055) cm per plant (CV = 0.37). Root length ratio-upper/lower ranged from 1.18 (#001) to 16.5 (#079) (CV = 0.62, all *p* < 0.01; Table 2). For the combined data of the 171 genotypes, more than 70% of the root length was in the 0–20 cm soil layer (Table 2, Figure 2).

Root diameter data showed that soybean plants had relatively fine roots with an average root diameter < 0.5 mm for all genotypes (Table 2). Most of the root length came from coarse roots (> 0.5 mm diameter class), accounting for 65% of the total root length. Root length (fine) contributed 35% to the total root length (Table 2).

#### 2.1.3. Correlation among Traits

A subset of nine traits, including eight root traits and one shoot trait (shoot mass), with CV ≥ 0.3 (Table 2), were selected for Pearson’s correlation analysis to identify relationships among the traits. Most of the selected traits had strong correlations with the other traits (Table 3). Total root length, root mass, shoot mass, and root length ratio–upper/lower were strongly associated with all other traits (mostly *p* ≤ 0.01) except for the specific root length.

For example, the total root length was positively correlated with root length (upper) (R^2^ = 0.912; Figure 4d), root length (lower) (R^2^ = 0.752; Figure 4d), root length (fine) (R^2^ = 0.352; Figure 4c), and root length (coarse) (R^2^ = 0.997; Figure 4c) (all *p* ≤ 0.01; Table 3). Root mass was positively correlated with total root length, root lengths (upper/lower), root lengths (coarse/fine), and negatively correlated with specific root length (all *p* ≤ 0.01; Table 3). Shoot mass was positively correlated with total root length (R^2^ = 0.669; Figure 4a) and root mass (R^2^ = 0.646; Figure 4b) (all *p* ≤ 0.01; Table 3). Root length (upper) was positively correlated with root length (lower) and root lengths (coarse/fine) (all *p* ≤ 0.01; Table 3). Specific root length was negatively correlated with total root length and shoot mass (Table 3). There were strong negative correlations between the root length ratio-upper/lower and all other traits except for the specific root length (Table 3).

#### 2.1.4. Root Trait Variability

Eight root traits and shoot mass with CVs ≥ 0.3 (Table 2) were included in the PCA. Two principal components (PCs) were identified with eigenvalues >1, capturing 78.9% of the total variation in root system architectural traits across the 171 soybean genotypes (Table 4). The first component (PC1) represented 66.2% of the variability and influenced by most root traits (total root length, root mass, root length-upper/lower, root lengths in diameter coarse, root lengths in diameter fine, root length ratio-upper/lower), and shoot mass (Table 4, Figure 5). PC2 represented 12.6% of the total variation and was influenced by the specific root length (Table 4, Figure 5).

Genotype distribution based on PCA regression scores of the nine selected traits is shown in a biplot (Figure 5), exhibiting clear separation of the three root size categories and a positive contribution of all selected traits, except for the specific root length and root length ratio (upper/lower) (Figure 5). Of the selected nine traits, root length, root mass, root length (upper), and root length (coarse) contributed the most to root size. The genotypes with extreme traits (outliers) are shown in the biplot (genotype #030, #054, #055, #071, #079, #153, #161 and #165) (Figure 5). In the PCA biplot based on the three source categories, genotypes #071, #079, #153, #161, and #165 also had extreme traits (outliers) (Figure S2). The same outer genotypes with extreme traits in two biplots should be selected for further study.

#### 2.1.5. Genotype Homogeneous Grouping Based on Root Trait Variation

An agglomerative hierarchical clustering (AHC) dendrogram was constructed using the Euclidean distance as the interval measurement on nine selected traits with CVs ≥ 0.3 and exhibits large diversity in traits among 171 soybean genotypes (Figure 6). The 171 soybean genotypes were separated into five main clusters/groups (G1 to G5), revealing variation in the degree of homogeneity among tested genotypes. Representative genotypes from each group were selected and used in further studies. Group 1 contained 56 genotypes, including 52 with a large root system and four with a medium root system. Group 2 contained one genotype (genotype #165) with a medium root system. Group 3 contained 13 genotypes with a large root system, 39 with a medium root system, and 38 with a small root system. Group 4 contained one genotype with a medium root system and 20 with a small root system. Group 5 contained three genotypes (genotype #030, #055 and #151) with a large root system. These results demonstrate that genotypes from the same root size were not always clustered into the same or closer group(s).

### 2.2. Validation of Root Characters in Rhizoboxes

#### 2.2.1. Phenotypes in Rhizoboxes

Three genotypes with putative differences in root system size were selected in a follow-up validation experiment. Genotypes #054 and #071 had small root systems, and #055 had a large root system (Figure 7). Large variation was found for the total root length, root mass, specific root length, root–shoot mass ratio, hypocotyl length, shoot height, shoot mass, and five local root traits (Table 5). There was no significant difference in root diameter (Table 5). Genotype #055 had the longest total root length and greatest root mass and shoot mass relative to #054 and #071, confirming the observations in the semi-hydroponic system (Table 5, Figure 8). From the seedling to flowering stage, genotype #055 produced significantly more roots than the other two genotypes (Figure 9). Genotype #055 (large root system) had flowered earlier than #054 and #071 (small root system) (Figure 9).

At 38 DAS, the root length (upper) significantly differed in the three genotypes (Table 5). The total root length ranged from 36.6 m per plant in #054 to 74.0 m per plant in #055 (Table 5). Genotype #055 had 1.8-fold and 1.2-fold more root mass than #054 and #071, respectively (Table 5, Figure 8c). Genotype #055 had 1.9-fold and 1.5-fold more root length (coarse) than #054 and #071 (Table 5). At all assessment times, most of the traits were strongly correlated (*p* ≤ 0.01; Table 6). Root mass and hypocotyl length were strongly associated with all other traits except for the rooting depth, specific root length, root–shoot mass ratio, and shoot height (mostly *p* ≤ 0.01; Table 6). For example, root mass had positive correlations with root length, root length (upper), and root length (coarse). Root length ratio (upper/lower) had negative correlations with root mass, root–shoot mass ratio, and root length (lower). The experiment confirmed that phenotypic variation in root size at the seedling stage (39 DAS) in a phenotyping study using the semi-hydroponic system was reproducible at the seedling stage (38 DAS) in a validation experiment using rhizoboxes.

At 81 DAS, the three genotypes significantly differed for root mass, total root length, shoot mass, root lengths (upper/lower), and root lengths (coarse/fine) (Table 5). In particular, genotype #055 had double the total root length and root length (fine) of #054 and #071 (Table 5). Genotype #055 also had 2.1-fold and 1.3-fold more root mass (Table 5, Figure 8d) and 1.5-fold and 1.2-fold more shoot mass than #054 and #071, respectively (Table 5, Figure 8b). At all assessment times, most root traits were strongly correlated (*p* ≤ 0.01; Table 6). For example, root length and root mass had strong correlations with shoot mass, root lengths (upper/lower), and root lengths (coarse/fine) (*p* ≤ 0.01; Table 6). The root length ratio (upper/lower) was not significantly correlated with other traits (Table 6).

At 160 DAS, root mass, hypocotyl length, shoot height, and shoot mass significantly differed between the three genotypes (Table 5). Genotype #055 had 1.9-fold and 5-fold more root mass, 1.9-fold and 3.6-fold more shoot mass, and 1.9-fold and 3.6-fold more shoot height than #054 and #071, respectively (Table 5). The three genotypes showed no significant difference in seed yield, although genotype #054 had more seeds and pods than the other two genotypes (data not presented).

#### 2.2.2. Correlations in Root and Growth Parameters between Experiments

Correlations were observed for the same traits in the semi-hydroponic system and the rhizobox system (38 DAS), with strong correlations at the 0.01 significance level for total root length, shoot mass, and root length (upper), and at the 0.05 significance level for hypocotyl length, shoot height, and root length (coarse) (Table 7a). However, there were no significant correlations for root diameter, root mass, specific root length, root–shoot mass ratio, root length (lower), root length (fine), or root length ratio (upper/lower), suggesting phenotypic plasticity in these traits at the seedling stage (Table 7a).

Data collected in rhizoboxes (81 DAS) were correlated with data for the same traits collected from the semi-hydroponic system, with strong correlations at the 0.01 significance level for root length, shoot height, and root lengths (upper/lower), and at the 0.05 level for hypocotyl length (Table 7b). However, there were no significant correlations for rooting depth, root mass, specific root length, shoot mass, root lengths (coarse), root lengths (fine), or root length ratio (upper/lower), suggesting phenotypic plasticity in these traits at the reproductive stage (Table 7b).

Data collected in rhizoboxes (160 DAS) were correlated with data for the same traits collected from the semi-hydroponic system, with strong correlations at the 0.01 significance level for root mass, root–shoot mass ratio, and shoot mass, and at the 0.05 significance level for hypocotyl length and shoot height (Table 7c).

## 3. Discussion

### 3.1. Variation among Root Architecture Traits

Studying root phenomics is increasingly becoming a crop-breeding strategy [21,40,41]. In the present study, among the measured root traits, the maximal root depth, total root length, root mass, root length (coarse), and root length (fine) significantly differed among the tested genotypes (Table 2), indicating the importance of these root traits in root system function. Studies have shown that these root traits are most important for water and nutrient acquisition [15,42,43,44]. Soybean selection and breeding programs mainly focus on aboveground traits and yield, with the impact on root traits often ignored [27,41]. Genotypes #54 (NG6255), #71 (Xudou 16), and #79 (NJP038) ranked in the small-root-system genotypes for total root length and root mass (Table S2, Figure 2). Genotypes #030 (Graham), #055 (Aijiaozao), and #151 (NJP530) ranked in the largest-root-system genotypes in terms of total root length and root mass (Table S2, Figure 2). The vigorous root system of these soybean genotypes enhances the acquisition of deep soil nutrients and water [3,45]. Studies have confirmed that modern soybean cultivars have larger root systems than older cultivars [46,47] due to breeding for increased grain yield. However, both root system architecture commonly appeared in newly developed cultivars/lines in our study, indicating less selection of this type of root traits.

Total root length is an important trait affecting root mass [1,48]. In this study, genotypes with the largest root systems had seven times more total root length and 12 times more root mass than genotypes with the smallest root systems (Table 2). There was a positive trend between root length, root mass, and shoot mass in both the semi-hydroponic system and rhizoboxes (Table 3 and Table 6). Total root length is an important factor affecting rooting depth [48]. Some studies have shown that the number of metaxylem elements in roots is correlated with drought tolerance in soybean [1,49]. Soybean genotypes with large root systems developed more metaxylem elements under drought stress, thus improving their resistance to stress [1].

As a leguminous species, soybean plants can form nodules to use nitrogen from the air on the cost of plant investment of carbon. Root system architecture could be altered during the nodulation [50], although some studies claimed no effect on the total root length of soybean following rhizobium inoculation [51]. The large phenotyping experiment was carried out under a semi-hydroponic environment, which was not suitable for the inoculation of rhizobium. The rhizoboxes experiment would have included rhizobium inoculation. However, inoculation was excluded to create non-inoculation conditions as in the semi-hydroponic system so that the root morphological traits of the selected three genotypes could be validated to confirm consistent genotype ranking in some important root traits (1) under both different growth conditions, (2) at early growth stages in both experiments, and (3) at different growth stages in the same soil environments. To avoid complexity involving inoculation treatments, adequate nitrogen was supplied to both experiments. The effects of rhizobium inoculation on soybean root configuration will be considered in our follow-up experiments through screening suitable rhizobium species and strains. Soybeans can form a symbiotic combination with rhizobium in a soil environment, which may be beneficial to improve nitrogen efficiency.

In this study, significant differences occurred between the soybean genotypes for the root lengths of coarse roots and fine roots. The genotype with the largest root system had seven times more root length (fine) and nine times more root length (coarse) than the smallest root system (Table 2). There were positive correlations between total root length, root length (coarse), and root length (fine) in both the semi-hydroponic system and rhizoboxes (Table 3 and Table 6), indicating that soybean genotypes with larger root systems had more coarse and fine roots than genotypes with smaller root systems. Fine roots constitute most of the root surface area and root length in cacao (*Theobroma cacao* L.) and played a leading role in water and nutrient absorption [44]. Some studies have shown that coarse roots are related to waterlogging tolerance in soybean. Soybean genotypes with more root length of the coarse roots tolerated waterlogging better than genotypes with fewer coarse roots [52]. Therefore, genotypes with larger root systems could be selected as candidate parents.

### 3.2. Genotype Selection Based on Root Trait Implications for Soybean Breeding

This study measured 18 traits including 14 root-related traits and four shoot traits (13 global traits and five local traits). A large variation in these traits was identified among the 171 soybean genotypes. Nine traits had CV ≥ 0.3, five of which were local traits (Table 2). Significant variation occurred in the local root traits of the tested soybean genotypes, especially for root length (lower) and root length (fine), which may be useful for breeding programs. Strong correlations between local traits and some global traits were identified in the Pearson’s correlation matrix (*p* ≤ 0.01; Figure 4, Table 3). Evidence of strong correlations between plant dry mass (shoot and root mass) and several local traits, including root length (upper) and root length (coarse), demonstrate that local traits are linked to plant growth strategies [53,54,55]. Studies have shown that crops with strong stress tolerance tend to have luxuriant root systems and high respiration rates in fine roots, which most likely reflects the metabolic activities related to nutrient absorption and assimilation [43,56,57].

Among the 171 genotypes, 92 genotypes had 40 to 50 days to anthesis with 35% being the large root system and 40% being the small root system (Table S1). Among the 68 large-root-system soybean genotypes, the days to maturity of 57% soybean genotypes was more than 100 days. We found that when genotypes had similar anthesis days, large-rooted genotypes had longer maturity days than other genotypes and absorbed more nutrients to meet the nutritional needs of the aboveground part during the mature period. This phenomenon may be controlled by some genes and needs further study.

Genotypes with a similar root system size (in terms of total root length and root mass) may distribute roots differently in the soil profile, as measured by root lengths (coarse/fine) and root lengths (upper/lower), mirror traits for total root length (Figure 3, Figure 4 and Figure 5). Several recent studies in soybean and maize demonstrated that genotypes with luxuriant lateral roots had better root growth deeper in the profile, phosphorus uptake, and yield in infertile soil than those with fewer lateral roots [43,58]. For example, under phosphorus limited conditions, large root system genotypes generally had high phosphorus utilization efficiency, and small root system genotypes had low phosphorus utilization efficiency (unpublished data). Differences in salt tolerance among soybean genotypes of different root system sizes reflect a potential gene regulation mechanism and remain to be further explored.

Through the PCA and AHC, some genotypes with extreme traits (outliers) were screened (Figure 5, Figure 6, and Figure S2). Genotypes #054 (NG6255), #071 (Xudou 16), and #079 (NJP038) had small root systems, genotypes #161 (Tongdou 2006) and #165 (Huaidou 10) had medium-sized root systems, and genotypes #030 (Graham), #055 (Aijiaozao) and #153 (NJP553) had large root systems. Some studies showed the special advantages of the individual genotype tested here. The large root system genotype #001 (Kefeng 1 Hao) is resistant to aluminum toxicity, and its seeds are resistant to storage [23,30]. Genotype #014 (Sidou 520) is an early-maturing summer soybean with strong lodging resistance [59]. Genotype #042 (Xudou 13) is high yielding and has strong drought resistance and high protein content [60]. The medium root system genotype #165 (Huaidou 10) is high yielding [61]. The seeds of small root system genotypes #092 (NJP133) and #112 (Vance) are resistant to storage [23]. These differences between genotypes and root system size provide a reference for the selection of candidate parents.

### 3.3. Validation of Root Characters in Soil

Using the established semi-hydroponic phenotyping system [31], this study elucidated phenotypic variability in numerous root morphological traits among 171 genotypes of soybean from different geographical sources, which are likely related to the ability of different genotypes to respond to the environment to optimize resource acquisition [19]. Attempts have been made to incorporate the digital root images acquired in the semi-hydroponic system, automated high-throughput computing, and collaboration platforms, such as optical character recognition (OCR) [62] to analyze crop root phenomics, particularly root length (fine).

Three genotypes (#054, #055, and #071) with putative differences in root system size were selected in a follow-up validation experiment using rhizoboxes filled with river sand soil. The large root system genotype, #055 (Aijiaozao), is a direct parent of Chinese soybean cultivars [63], and it is also an old variety with good adaptation in Hubei province and the southern Huaihe River region [64]. The small root system genotype #071 (Xudou 16) is a medium-maturing summer soybean cultivar with high and stable yield in the northern Huaihe River region [65]. The genotype #054 (NG6255) is a high-yield and good lodging resistance line introduced from USA. The correlation analysis of the semi-hydroponic system and the rhizobox system (38 DAS) showed significant positive correlations between total root length, root length (upper) (*p* ≤ 0.01), and root length (coarse) (*p* ≤ 0.05) (Table 7a), indicating that the total root length at the soybean seedling stage mainly comprises root length (upper) and root length (coarse). The correlation analysis of the semi-hydroponic and the rhizobox systems (81 DAS) showed significant positive correlations between total root length and root lengths (upper/lower) (all *p* ≤ 0.01; Table 7b), indicating that the total root length at the soybean reproductive stage mainly comprised root lengths (upper/lower). The correlation analysis showed significant positive correlations between root mass, root–shoot mass ratio, and shoot mass at soybean maturity between the semi-hydroponic and the rhizobox systems (160 DAS) (all *p* ≤ 0.01; Table 7c), indicating that these traits were intrinsic. Significant positive correlations between hypocotyl length and shoot height between the semi-hydroponic and the rhizobox systems at various growth stages (all *p* ≤ 0.05; Table 7) confirmed that these shoot traits in later growth stages in soil can be predicted from the early growth in the semi-hydroponic system. However, several root traits including root diameter and root length ratio (upper/lower) (Table 7) were not significantly correlated under the two experimental conditions, which may indicate the plasticity of these traits in response to growth environments [32].

The experiment confirmed that phenotypic variation in the root system size at the seedling stage using the semi-hydroponic system in this phenotyping study was reproducible across the whole soybean growing period in the validation experiment using rhizoboxes. During the whole growing period, root growth positively correlated with the genotypic sequencing of some important root traits, indicating that the semi-hydroponic phenotypic system provides simple and reliable growing conditions for studying root phenotypes. A series of early studies on wild narrow-leafed lupin roots also revealed the susceptibility of the semi-hydroponic phenotypic system through repeated experiments in different environments (including field environments) [32,33,66]. Some studies have investigated the phenotypic correlation of roots in controlled and soil environments. For example, large root system genotypes #001 (Kefeng 1 Hao), #006 (Nannong 86-4), and #014 (Sidou 520) had drought tolerance, #030 (Graham) and #042 (Xudou 13) had medium drought tolerance, and #049 (t821058), #051 (NT821060), #055 (Aijiaozao), and #067 (Suxian 21) had drought susceptibility [40]. In another study, small root system genotypes #002 (Nannong 88-31) and #079 (NJP038) had high drought tolerance, #108 (Jilin 31) had drought tolerance, #040 (Tongdou 7) and #112 (Vance) had medium drought tolerance, #052 (Zhechun 3 Hao), #054 (NG6255), and #071 (Xudou 16) had drought susceptibility [64].

However, there are limitations in converting phenotypic data from the seedling stage obtained from semi-hydroponic systems to soybean breeding programs. Roots may have specific responses in different growth environments, and phenotypic plasticity is important for searching for soil resources [18]. Therefore, the phenotypic system needs to produce a reliable ranking of root characteristics, and the selection of growth medium needs to be considered carefully. The development of modern root imaging technology and root simulation modeling can be integrated into the phenotypic platform.

## 4. Materials and Methods

Two glasshouse experiments were conducted in a temperature-controlled glasshouse at Northwest A&F University, Yangling (34°16′ N, 108°4″ E) in 2018 and 2019, respectively. The first experiment using a semi-hydroponic phenotyping system [31] characterized root trait variability among 171 soybean (*Glycine max* L. Merr.) genotypes. The second experiment validated root properties of three genotypes selected from the semi-hydroponic system, using 1.5 m deep rhizoboxes filled with soil.

### 4.1. Soybean Genotypes

The semi-hydroponic experiment used 171 soybean genotypes (including 68 genotypes from the northern Huaihe River region, 75 genotypes from the southern Huaihe River region, and 28 parental lines, which had been previously tested in the field in the Yangtze and Huaihe River regions in eastern China [64]) (Table S1). Three genotypes, #054, #055, and #071, with contrasting root system size were selected for the validation study using soil-filled rhizoboxes. Seeds were provided by the National Center for Soybean Improvement, National Key Laboratory for Corp Genetics and Germplasm Enhancement, Soybean Research Institute, and Nanjing Agricultural University in Nanjing, China.

### 4.2. Experimental Design, Layout, and Maintenance

#### 4.2.1. The Semi-Hydroponic Phenotyping System

**Figure 1:**
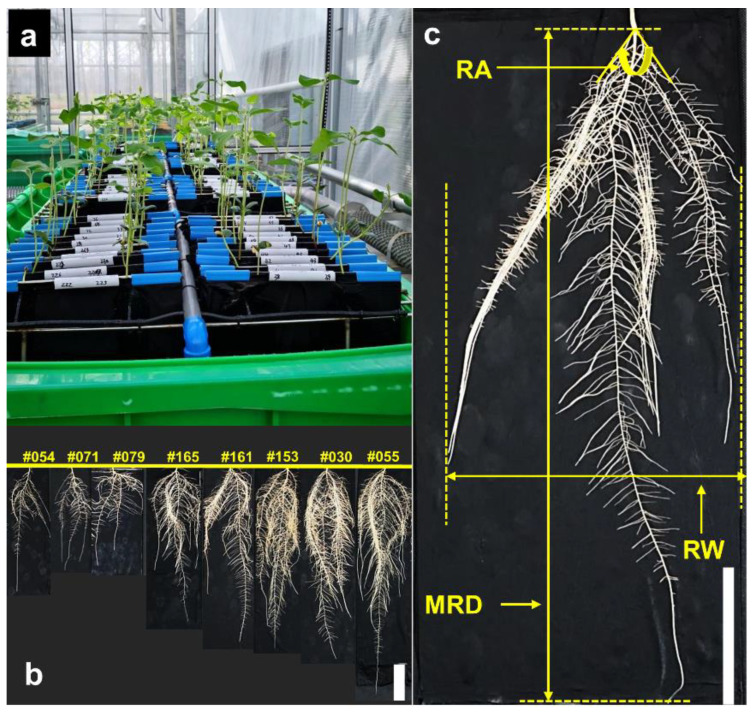
Layout of soybean plants grown in a semi-hydroponic phenotyping platform at 19 days after sowing (DAS) (**a**), and example images of root systems of eight selected genotypes at 39 DAS (**b**). Root angle (RA), root width (RW) and maximal root depth (MRD) of the soybean root system grown in a semi-hydroponic phenotyping platform (**c**), were measured at 39 DAS. White bar = 10 cm.

**Figure 2:**
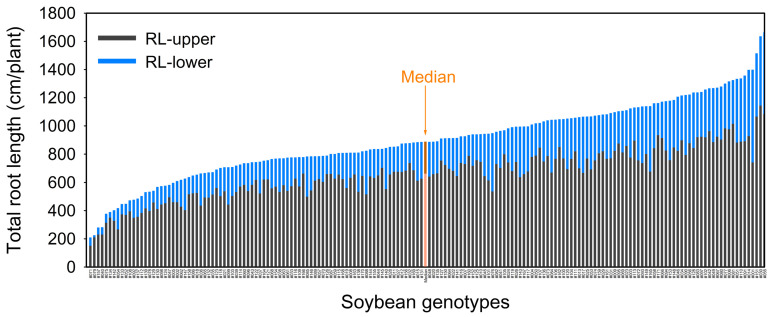
Stacked vertical barplot showing genotypic variation in root length of 171 soybean genotypes grown in a semi-hydroponic phenotyping platform 39 days after sowing. Median values were plotted for all genotypes. Both RL-upper (root length in 0–20 cm soil layer) and RL-lower (root length below 20 cm soil depth) are presented (ordered from shortest to longest average total root length).

**Figure 3:**
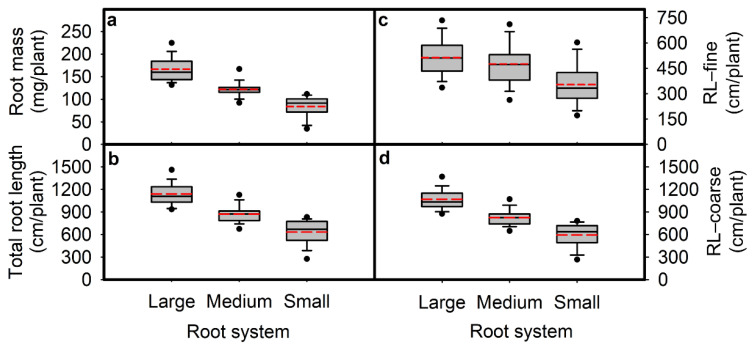
Root mass (RM; (**a**)), total root length (RL; (**b**)), root length in diameter fine (RL-fine; (**c**)) and root length in diameter coarse (RL-coarse; (**d**)) for genotypes in three root size categories large (68 genotypes), medium (45 genotypes), and small (58 genotypes). The 171 genotypes were grown in a semi-hydroponic phenotyping platform 39 days after sowing. Significant differences are shown for the three root size categories (*p* ≤ 0.05). The whiskers, box, and dot are determined by the 5th and 95th percentiles, 25th and 75th percentiles, and the 1st and 99th percentiles, respectively. The line and dashed inside the box marks are the median and mean, respectively.

**Figure 4:**
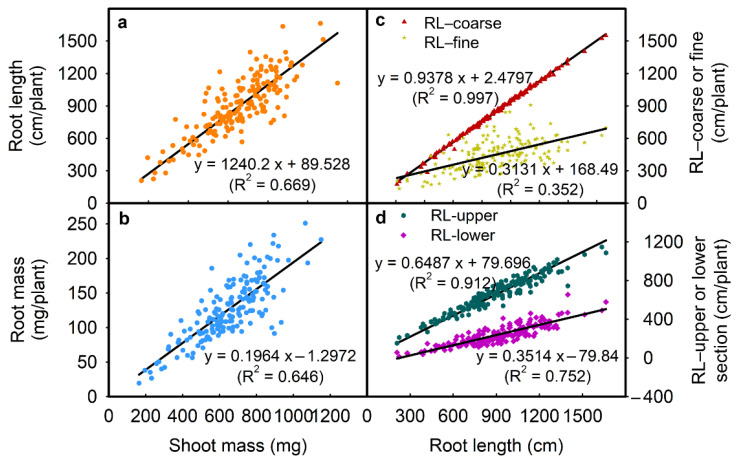
Correlation between (**a**) shoot mass and root length, (**b**) shoot mass and root mass, (**c**) root length and RL-coarse or RL-fine, and (**d**) root length and RL-upper or RL-lower in 171 soybean genotypes grown in a semi-hydroponic phenotyping platform 39 days after sowing. Each dot represents the mean values of three replicates.

**Figure 5:**
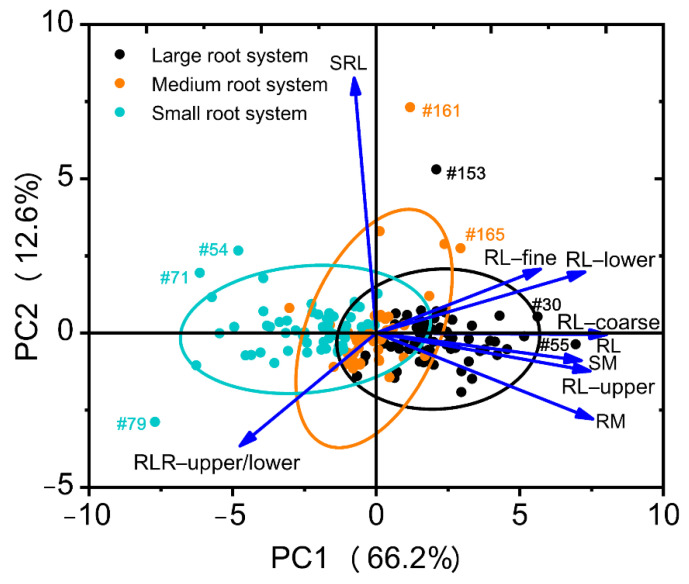
Principal component analysis of nine selected traits with CVs ≥ 0.3 across 171 soybean genotypes grown in a semi-hydroponic phenotyping platform 39 days after sowing. The position of each trait is shown for PC1 vs. PC2 representing 78.9% of the total variability. The genotypes with extreme traits (outliers) are indicated by genotype number.

**Figure 6:**
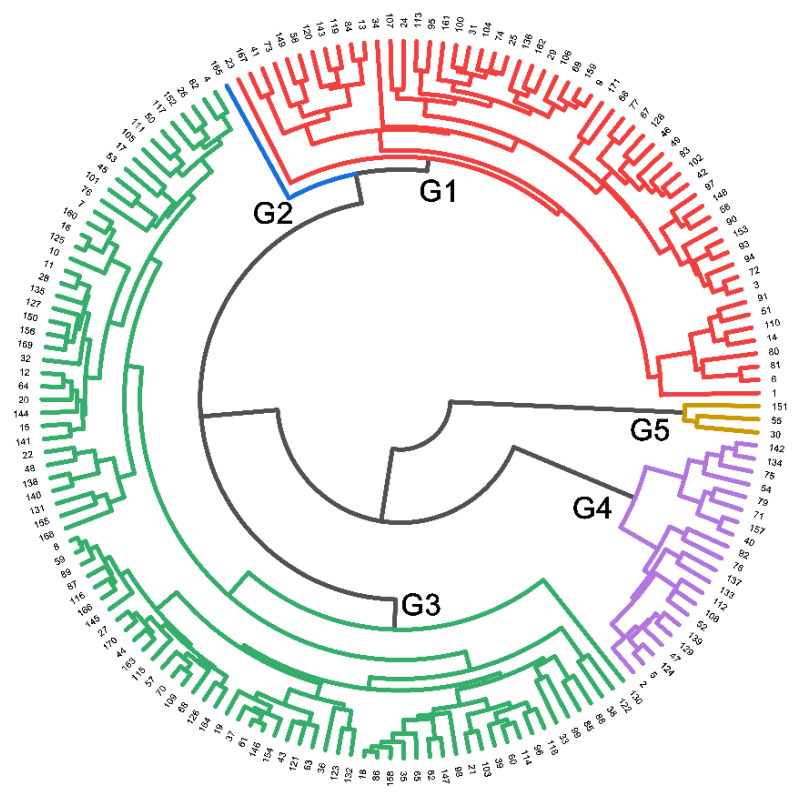
Dendrogram of agglomerative hierarchical clustering (AHC) using the average linkage method with Euclidean distance as the interval measurement on nine selected traits with CVs ≥ 0.3. The 171 soybean genotypes were assigned to five groups (G1 to G5). The nine traits are the same as those used for PCA in Table 4 (the semi-hydroponic system).

**Figure 7:**
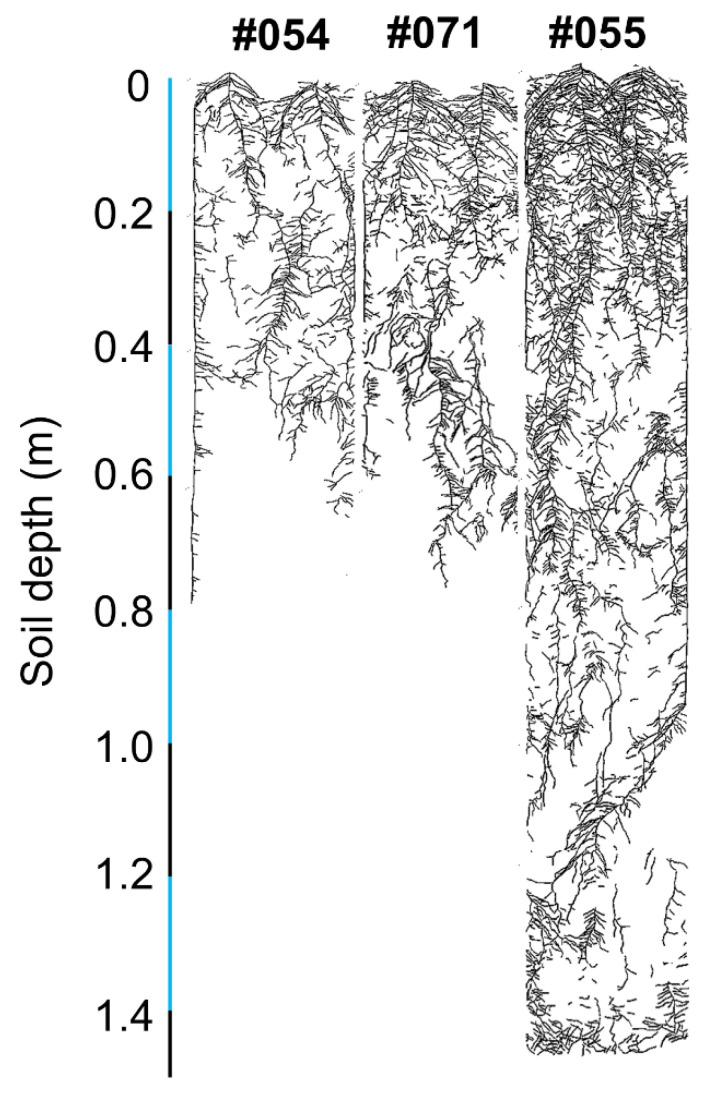
Scanned image of traced root systems on the glass panels of the rhizoboxes for soybean genotypes #054, #055, and #071 at 93 days after sowing.

**Figure 8:**
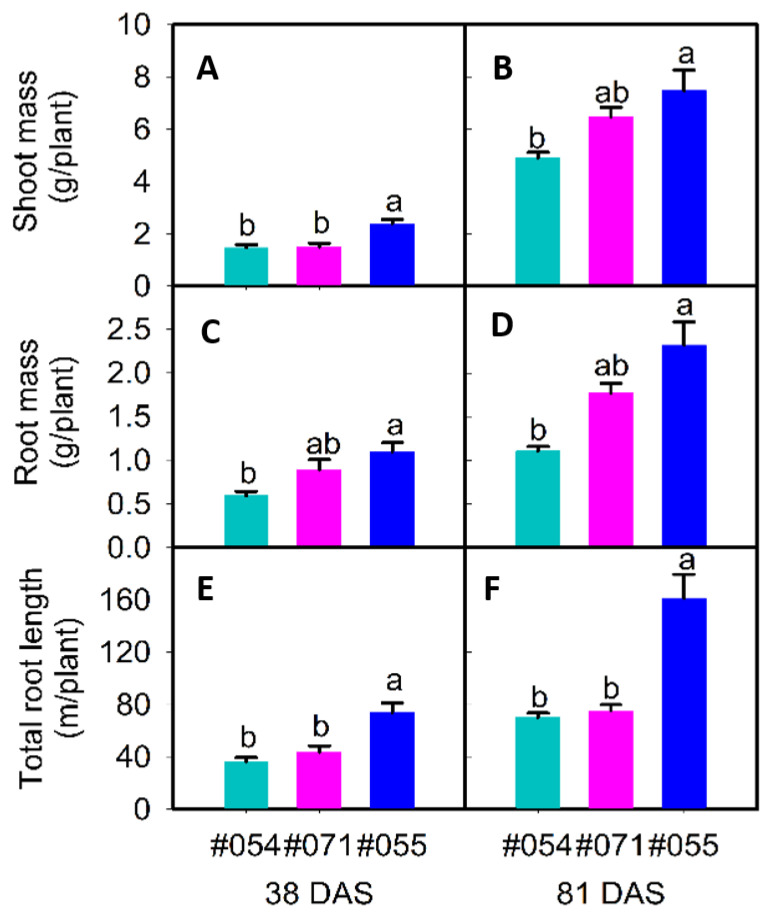
Shoot mass (**A**,**B**), root mass (**C**,**D**), and total root length (**E**,**F**) of three soybean genotypes (#054, #055, and #071) 38 days after sowing (DAS) ((**A**,**C**,**E**), R1 growth stage except #054, which was about 44 DAS) and 81 DAS ((**B**,**D**,**F**), all genotypes at R5 growth stage) grown in soil-filled rhizoboxes. For each trait, mean data (+SE, *n* = 3) with different letters indicate significant difference among the three genotypes (*p* ≤ 0.05).

**Figure 9:**
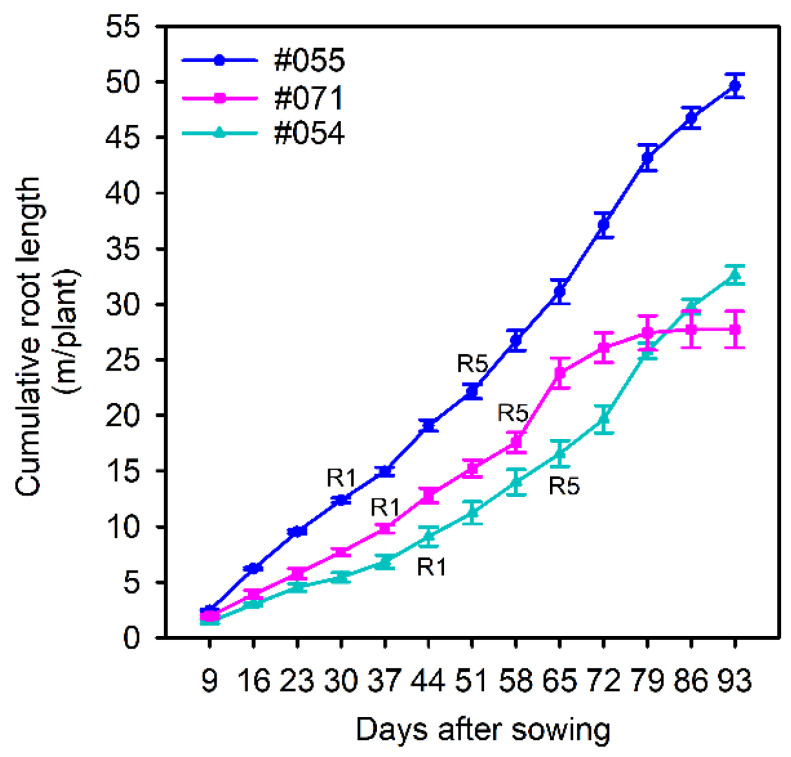
Cumulative root length of three soybean genotypes (#054, #055, and #071) from nine days after sowing (DAS) to 93 DAS (the rhizobox system). Genotype #054 and #071 have small root systems and #055 has a large root system (soybean growth stage: R1–time to flowering; R5–time to forming seed pods).

A semi-hydroponic phenotyping system (Figure 1a) [31] was used to characterize root trait variability in 171 soybean genotypes. Each system comprises of one 240 L wheelie bin, 20 growth units made of a transparent acrylic panel (250 × 500 mm, 4 mm thick) wrapped by black calico cloth, pump, and irrigation system, and supporting frames [31,67]. The black cotton cloth in the plant growth unit retains moisture through the automatic pumping system. Each bin was filled with 30 L nutrient solution containing (μM): K (1220), P (50), S (1802), Ca (600), Mg (200), Cu (0.2), Zn (0.75), Mn (0.75), B (5), Co (0.2), Na (0.06), Mo (0.03), Fe (40), and N (1000).

Soybean seeds were sown in two lots on two separate days to create a one-day difference in planting and subsequent harvest for ease of the process. Seeds were surface-sterilized in 10% H_2_O_2_ for 10 min and sown in pots filled with water-washed river sand. After six days, geminated seedlings with 2–3 cm long roots were carefully washed free of sands and transferred into the growth system. Two plants of two different genotypes were transplanted into each growth unit with 40 plants per bin system. Four plants of each genotype were planted in four replicate bins. Twenty-four bins were arranged in a randomized block design with six bins as one replicate. Buffer plants were used when required to ensure an equal number of plants assigned to each bin. The average daily temperature during the experimental period was approximately 25/15 °C (day/night). The water supply time was controlled by a timer. Constant water supply was given during the first three days after transplantation. Then, the water was supplied with 10 min on and 5 min off for the remainder of the experiment using a timer to control the pump/irrigation system. Each bin was randomly repositioned once a week. The nutrient solution was refreshed weekly.

#### 4.2.2. Soil-Filled Rhizoboxes

A randomized block design comprising three soybean genotypes (#054, #055, and #071) selected from the semi-hydroponic experiments was conducted with three harvests and three replicates. Each rhizobox served as a replicate for a total of 27 rhizoboxes (25 cm long × 5 cm wide × 150 cm deep). Rhizoboxes were constructed from polyvinyl chloride (PVC) with one glass side that was covered with a black PVC sheet to block the light. The rhizoboxes were placed on steel stands at a 30° angle. Each rhizobox was filled with 28.5 kg of air-dried distill water washed river sand (<2 mm) and irrigated with 5 L of nutrition solution as the same in the semi-hydroponic system. Nutrient solution was applied weekly. Four seeds were surface-sterilized and sown on 16 May 2019 in a row close to the glass wall of each rhizobox. Seedlings were thinned to two plants per rhizobox at the cotyledon stage. Plants were watered twice a day (300 mL each time) when the nutrition solution was not applied. Rhizoboxes were randomly repositioned once a week. The average daily temperature during the experimental period was approximately 30/20 °C (day/night).

### 4.3. Measurements and Assessments

#### 4.3.1. The Semi-Hydroponic Phenotyping System

Plants were assessed 39 days after sowing (DAS). At harvest, the values of maximum physical height of plant shoots, leaf number, and taproot length per plant were measured manually. Shoot height was measured with a ruler from the cotyledon node to the peak of the main stem. The growth panels were taken out of the bin and placed on a flat bench with a black background. The cloth was removed from the growth panel to expose the root systems for photographing using a camera (Sony LICE-7, SONY CORP, Tokyo, Japan) installed above the workbench. Shoots and roots were separated after photographing. Maximal root width (the maximal horizontal width of a root system), root angle (maximal growth angle between two outermost lateral roots) (Figure 1c), and hypocotyl root length were measured using ImageJ (1.51j8, Wayne Rasband, National Institutes of Health, USA). Shoot dry weight (i.e., shoot mass) was determined after drying in an air-forced oven at 75 °C for 72 h. Root subsamples were collected by cutting the root system into 10 cm sections from the base, which were stored in a 4 °C refrigerator until scanning for morphological and architectural measurements. After scanning, root subsamples from the same plant were collected as one root sample and dried in an oven to obtain root dry mass.

#### 4.3.2. Soil-Filled Rhizoboxes

Plants were harvested 38 days after sowing (DAS), 81 DAS, and 160 DAS. The number of visible roots via the glass wall was recorded every seven days after the VC stage (unifoliolate leaves completely expanded, 9 DAS) until harvest. For each weekly measurement, the black PVC cover was removed, and the glass wall was covered with transparent plastic film (A4 paper size) to trace the visible new roots using a waterproof permanent black marker pen. After removing the transparent film from the glass wall, all visible new roots were also traced onto the glass wall before covering the glass wall with the black PVC cover. The root growth data were obtained by scanning the film with a desktop scanner (Epson Perfection V800, Long Beach, CA, USA) on each mapping day.

At harvests, the maximum physical height of plant shoots and hypocotyl length was measured manually. The black PVC cover and glass wall were removed from the rhizobox to expose the visible root systems for photographing using a camera installed above the workbench with the method similar to the semi-hydroponic system. After photographing, the shoots and roots were separated at the soil surface. Shoot dry mass was determined after drying in an air-forced oven. Roots subsamples were collected by cutting the root system into 10 cm sections from the base. The subsamples were washed on a 1.4 mm sieve with water. The roots were stored in a 4 °C refrigerator until scanning for morphological and architectural measurements. The method of measuring root dry mass is the same as the semi-hydroponic experiments.

### 4.4. Root Scanning and Root Image Analysis

Root section samples from both experiments and root tracing films from soil-filled rhizoboxes (except for root samples harvested at 160 DAS) were scanned in grayscale at 300 dpi using a desktop scanner (Epson Perfection V800, Long Beach, CA, USA). The root images for each 10 cm segment were analyzed using WinRhizo Pro (v2009, Regent Instruments, Montreal, QC, Canada). Imaging was analyzed using the debris removal filter of discounting objects < 1 cm^2^ with a length–width ratio < 8 in the image. Root morphology data, such as total root length, average root diameter for each root section, and root length for different diameter classes were generated in the WinRhizo program. Root length diameters < 0.5 mm were categorized as fine roots, and ≥ 0.5 mm were categorized as coarse roots. The following root traits were calculated from the measured data:

Specific root length (SRL) = total root length/root dry mass;

Root tissue density (RTD) = total root dry mass/root volume;

Root–shoot mass ratio (RSM) = total root dry mass/shoot dry mass;

Root length ratio (upper/lower) = root length in top 20 cm section/root length below 20 cm soil depth.

The 18 root traits were divided into two general categories (semi-hydroponic system): 13 global traits (whole plant level) and five local traits (Table 1). Global traits refer to the whole root system and whole shoots, and local root traits refer to roots in different depths and diameter classes.

### 4.5. Data Treatment and Analysis

One-way ANOVA was conducted using SPSS Statistics 22 (IBM Corporation, Somers, NY, USA) for significant differences among the tested genotypes for each trait. Figures were plotted using SigmaPlot 12.5 (Systat, USA) and Origin 2021 (OriginLab, Northampton, MA, USA). Correlations were considered statistically significant at *p* ≤ 0.05 (*) or *p* ≤ 0.01 (**). Traits with coefficients of variation (CV) ≥ 0.3 were selected for principal component analysis (PCA) to identify determinants of root architecture variability across genotypes [68]. Hierarchical cluster analysis was used to determine the variance among the selected root traits and homogeneous groups among the genotypes using the Euclidean distance method.

## 5. Conclusions

The quality root data and consistent ranking of genotypes in root traits demonstrated the reliability of the semi-hydroponic system in phenotyping root trait variability among a large set of soybean genotypes. Substantial variation in root-related traits across 171 soybean genotypes was identified and validated in selected three genotypes with contrasting root system properties. Genotypes with interesting root system architecture traits, including total root length, root mass, root lengths in diameter coarse, root lengths in diameter fine and trait–trait relationships, could be used for crossing with widely adapted cultivars in soybean breeding programs after further observations of their performance under field conditions. With the availability of the whole genome sequencing data of the soybean resources used in this study, we will conduct genome-wide association analysis study (GWAS) to identify loci and genes controlling specific root morphological traits. The eventual aims of these studies are to select and breed soybean cultivars with suitable root traits for enhanced adaptation to adverse environments and improved nutrient and water use efficiency.

## Figures and Tables

**Table 2:** Descriptive statistics of 18 measured traits (13 global and five local traits) in 171 soybean genotypes grown in a semi-hydroponic phenotyping platform assessed 39 days after sowing.

Trait	Minimum	Maximum	Median	Mean	Std. Dev.	CV	*p* Value
MRD	28.8	65.0	47.5	46.6	7.37	0.16	***<0.001***
RW	9.57	20.5	14.7	14.9	2.70	0.18	***<0.001***
RA	63.0	160	109	109	28.0	0.26	***<0.001***
RL	208	1663	885	898	331	**0.37**	***<0.001***
RD	0.20	0.47	0.42	0.42	0.03	0.07	***<0.001***
RM	19.4	251	124	127	55.8	**0.44**	***<0.001***
SRL	5.27	25.6	7.33	7.72	3.81	**0.49**	0.64
RTD	98.0	219	141	143	40.2	0.28	0.91
RSM	0.11	0.32	0.19	0.19	0.05	0.27	***<0.001***
LN	3.00	6.00	5.00	4.81	0.67	0.14	***<0.001***
HL	2.20	9.10	4.50	4.55	1.24	0.27	***<0.001***
SH	18.7	86.7	52.3	51.0	11.3	0.22	***<0.001***
SM	164	1150	663	652	213	**0.33**	***<0.001***
RL-upper	152	1146	664	662	226	**0.34**	***<0.001***
RL-lower	11.5	654	223	236	136	**0.58**	***<0.001***
RL-fine	134	908	448	450	208	**0.46**	***<0.001***
RL-coarse	175	1551	849	845	309	**0.37**	***<0.001***
RLR-upper/lower	1.18	16.5	3.27	3.59	2.23	**0.62**	***<0.001***

Traits with coefficients of variation (CVs) ≥ 0.3 appear in bold type. Probability values (*p* value) were based on a GLM multivariate analysis of 171 genotypes and appear in bold if ≤0.01 and italics if ≤0.05 (see Table 1 for trait descriptions and units). Std. Dev., standard deviation.

**Table 3:** Pearson’s correlation matrix for eight root traits (RL, SRL, RL-upper, RL-lower, RL-fine, RL-coarse, and RLR-upper/lower) and one shoot trait (SM) in 171 soybean genotypes grown in a semi-hydroponic phenotyping platform assessed 39 days after sowing.

-	RM	SRL	SM	RL- Upper	RL- Lower	RL- Fine	RL- Coarse	RLR-Upper/ Lower
RL	0.903 **	−0.023 ns	0.818 **	0.955 **	0.867 **	0.593 **	0.998 **	−0.469 **
RM	-	−0.322 **	0.803 **	0.868 **	0.774 **	0.505 **	0.900 **	−0.429 **
SRL	-	-	−0.101 ns	−0.055 ns	0.034 ns	−0.022 ns	−0.033 ns	−0.014 ns
SM	-	-	-	0.812 **	0.657 **	0.599 **	0.820 **	−0.404 **
RL-upper	-	-	-	-	0.680 **	0.509 **	0.951 **	−0.303 **
RL-lower	-	-	-	-	-	0.610 **	0.869 **	−0.650 **
RL-fine	-	-	-	-	-	-	0.608 **	−0.514 **
RL-coarse	-	-	-	-	-	-	-	−0.474 **

Traits with CVs ≥ 0.3 were included in the analysis (Table 2). Correlation is significant if *p* ≤ 0.01 (**); ns, no significance (see Table 1 for trait descriptions and units).

**Table 4:** Principal component analysis of nine selected traits with CV ≥ 0.3 and the proportion of variation in each principal component.

Traits	PC1	PC2
RL	0.976	−0.016
RM	0.917	−0.309
SRL	−0.083	0.883
SM	0.870	−0.106
RL-upper	0.907	−0.143
RL-lower	0.889	0.199
RL-fine	0.701	0.212
RL-coarse	0.978	−0.018
RLR-upper/lower	−0.585	−0.383
Eigenvalue	5.96	1.14
Variability (%)	66.2	12.6
Cumulative (%)	66.2	78.9

See Table 1 for trait descriptions and units.

**Table 5:** Mean values of 13 measured traits (eight global and five local traits) in three soybean genotypes (#054, #055, and #071) grown in soil-filled rhizoboxes at 38 days after sowing (DAS), 81 DAS, and 160 DAS. At 160 DAS, only root mass (RM), root–shoot mass ratio (RSM), hypocotyl length (HL), shoot height (SH), and shoot mass (SM) were measured.

Trait	Unit	38 DAS			81 DAS			160 DAS		
		#054	#055	#071	#054	#055	#071	#054	#055	#071
RM	g plant^−1^	0.59 b	1.10 a	0.89 ab	1.11 b	2.32 a	1.78 ab	6.70 b	12.5 a	2.36 c
RSM	-	0.41 b	0.46 ab	0.59 a	0.23 b	0.31 a	0.27 ab	0.30 ns	0.30 ns	0.20 ns
HL	cm	4.25 b	8.17 a	5.92 b	4.05 b	7.50 a	7.00 a	5.00 b	7.67 a	6.42 ab
SH	cm	98.0 ab	107 a	80.5 b	100 ab	114 a	88.5 b	108 ab	121 a	93.3 b
SM	g plant^−1^	1.48 b	2.38 a	1.50 b	4.90 b	7.47 a	6.48 ab	22.1 ab	41.7 a	11.6 b
RL	m	36.6 b	74.0 a	43.7 b	70.2 b	161 a	75.3 b	-	-	-
RD	mm	0.39 ns	0.41 ns	0.40 ns	0.44 ns	0.43 ns	0.42 ns	-	-	-
SRL	m g^−1^	6.17 ab	6.76 a	4.94 b	6.34 ab	6.93 a	4.24 b	-	-	-
RL-upper	m	36.0 b	71.0 a	40.4 b	62.7 b	124 a	60.7 b	-	-	-
RL-lower	m	0.32 b	3.04 a	3.31 a	7.54 b	37.3 a	14.6 b	-	-	-
RL-fine	m	30.5 b	62.7 a	36.2 b	57.4 b	134 a	62.1 b	-	-	-
RL-coarse	m	5.83 b	11.2 a	7.52 ab	12.8 b	27.5 a	13.2 b	-	-	-
RLR-upper/lower	m	116 a	25.9 b	12.4 b	16.5 a	3.39 b	5.17 b	-	-	-

For each trait, data with the same letter indicate no significant difference between cultivars (*p* ≤ 0.05, see Table 1 for trait descriptions), ns, no significance.

**Table 6:** Pearson’s correlation matrix for ten root traits and three shoot traits in three soybean genotypes (#054, #055, and #071) grown in soil-filled rhizoboxes at 38 days after sowing (lower left section) and 81 days after sowing (upper right section).

-	RL	RD	RM	SRL	RSM	HL	SH	SM	RL-Upper	RL-Lower	RL-Fine	RL-Coarse	RLR-Upper/Lower
RL	-	0.116	0.879 **	0.591	0.750 *	0.637	0.583	0.807 **	0.991 **	0.958 **	0.990 **	0.858 **	−0.385
RD	0.377	-	0.053	0.181	0.256	−0.014	0.187	−0.107	0.073	0.201	0.129	0.031	−0.033
RM	0.865 **	0.621	-	0.138	0.860 **	0.885 **	0.292	0.952 **	0.828 **	0.935 **	0.862 **	0.781 *	−0.545
SRL	0.504	−0.271	0.020	-	0.117	−0.161	0.769 *	0.065	0.651	0.425	0.602	0.451	0.108
RSM	0.012	0.408	0.459	−0.785 *	-	0.819 **	0.333	0.670 *	0.694 *	0.827 **	0.762 *	0.574	−0.552
HL	0.913 **	0.298	0.866 **	0.265	0.191	-	0.179	0.830 **	0.587	0.707 *	0.644	0.495	−0.332
SH	0.604	0.387	0.314	0.599	−0.477	0.479	-	0.203	0.595	0.522	0.649	0.247	−0.072
SM	0.949 **	0.330	0.762 *	0.604	−0.218	0.838 **	0.667 *	-	0.761 *	0.858 **	0.779 *	0.764 *	−0.509
RL-upper	0.998 **	0.356	0.833 **	0.554	−0.046	0.893 **	0.633	0.956 **	-	0.912 **	0.983 **	0.845 **	−0.286
RL-lower	0.596	0.450	0.850 **	−0.274	0.668 *	0.756 *	0.011	0.457	0.538	-	0.945 **	0.834 **	−0.575
RL-fine	0.998 **	0.336	0.844 **	0.528	−0.014	0.917 **	0.616	0.948 **	0.997 **	0.581	-	0.779 *	−0.380
RL-coarse	0.953 **	0.576	0.925 **	0.349	0.152	0.845 **	0.509	0.902 **	0.944 **	0.647	0.935 **	-	−0.338
RLR-upper/lower	−0.493	−0.253	−0.761 *	0.365	−0.770 *	−0.690 *	0.186	−0.314	−0.438	−0.903 **	−0.484	−0.513	-

Correlation is significant if *p* ≤ 0.05 (*) or *p* ≤ 0.01 (**) (see Table 1 for trait descriptions and units).

**Table 7:** Correlation of some important traits (ten root traits and three shoot traits) three soybean genotypes (#054, #055, and #071) between the semi-hydroponic system (39 days after sowing, DAS) and the data from rhizoboxes assessed (a) 38 DAS, (b) 81 DAS, and (c) 160 DAS. At 160 DAS, only root mass (RM), root–shoot mass ratio (RSM), and shoot mass (SM), were measured.

**Traits**	**(a) 38 DAS**	**(b) 81 DAS**	**(c) 160 DAS**
RM	0.591	0.665	0.901 **
RSM	−0.648	0.187	0.884 **
HL	0.690 *	0.670 *	0.672 *
SH	0.732 *	0.801 **	0.776 *
SM	0.887 **	0.511	0.900 **
RL	0.854 **	0.886 **	-
RD	0.004	0.310	-
SRL	−0.397	−0.563	-
RL-upper	0.831 **	0.851 **	-
RL-lower	0.299	0.866 **	-
RL-fine	0.179	0.202	-
RL-coarse	0.733 *	0.664	-
RLR-upper/lower	−0.196	0.195	-

Correlation is significant if *p* ≤ 0.05 (*) or *p* ≤ 0.01 (**) (see Table 1 for trait descriptions and units).

## Data Availability

Relevant data generated or analyzed during this study are included in this article and its supplementary information files. Other data are available upon request to the corresponding author.

## Supplementary Materials

The following are available online at https://www.mdpi.com/article/10.3390/plants10122781/s1, Table S1: List of the 171 genotypes of soybean (*Glycine max* L. Merr.) used in the phenotyping study using a semi-hydroponic system with three selected genotypes used in the validation experiment using rhizoboxes in this study. Information about adaptive regions, days to anthesis, and days to maturity (from seed emergence; VE-R1 stage), root system size, and drought tolerance is provided, Table S2: Soybean genotypes ranked in the top 15 (large values, indicated by ) or bottom 15 (small values, indicated by ) among 171 genotypes from the phenotyping experiment for total root length (RL), with some also ranked in the top/bottom 15 for other traits. Figure S1: Root mass (RM; a), total root length (RL; b), root length in diameter fine (RL-fine; c), and root length in diameter coarse (RL-coarse; d) for genotypes in three source categories NHHR (68 genotypes), SHHR (75 genotypes), and PL (28 genotypes) used in the phenotyping experiment. NHHR, northern Huaihe River region; SHHR, southern Huaihe River region; PL, parental lines. The 171 genotypes were grown in a semi-hydroponic phenotyping platform 39 days after sowing. Significant differences are shown for the three source categories (*p* ≤ 0.05). The whiskers, box, and dot are determined by the 5th and 95th percentiles, 25th and 75th percentiles, and the 1st and 99th percentiles, respectively. The line and dashed inside the box marks are the median and mean, respectively, Figure S2: Principal component analysis of nine selected traits with CVs ≥ 0.3 across 171 soybean genotypes grown in a semi-hydroponic phenotyping platform 39 days after sowing. The position of each trait is shown for PC1 vs. PC2 representing 78.6% of the total variability. The genotypes with extreme traits (outliers) are indicated by genotype number. NHHR, northern Huaihe River region; SHHR, southern Huaihe River region; PL, parental lines.

## Abbreviations

AHC: Agglomerative hierarchical clustering; GLM: General Linear Model; *p*: Probability; PCA: Principal component analysis.
