# Supplementary material for: Characterization of Root System Architecture Traits in Diverse Soybean Genotypes Using a Semi-Hydroponic System
Source: Plants (Basel). 2021 Dec 16;10(12):2781. doi: 10.3390/plants10122781 (PMC8707277; doi:10.3390/plants10122781)
Supplement: Supplementary file 1 [file plants-10-02781-s001.zip › plants-1467362-supplementary.pdf]

Supplementary data

Table S1. List of the 171 genotypes of soybean (*Glycine max* L. Merr.) used in the phenotyping study using a semi-hydroponic system with three selected genotypes used in the validation experiment using rhizoboxes in this study. Information about adaptive regions, days to anthesis and days to maturity (from seed emergence; VE-R1 stage), root system size, and drought tolerance is provided.

Code #	Name	Adaptive regions #	Days to anthesis (d) €	Days to maturity (d) €	Root system size £	Drought tolerance $
#001	Kefeng 1 Hao	Beijing	37.5	94.3	large	Drought tolerance
#002	Nannong 88-31	SHRR	42.3	102.0	small	Drought tolerance
#003	NJP023	NHRR	39.7	96.4	large	Unknown
#004	NJ90L-1	SHRR	45.9	104.9	medium	Drought tolerance
#005	DHP	Henan	37.8	95.7	small	Drought tolerance
#006	Nannong 86-4	SHRR	44.3	100.3	large	Unknown
#007	NT821101	NHRR	47.1	102.3	large	Drought tolerance
#008	Nannong 493-1	SHRR	46.8	104.0	medium	Drought tolerance
#009	Nannong 1138-2	SHRR	44.9	99.3	large	Unknown
#010	NG94-156	SHRR	42.8	103.3	large	Drought tolerance
#011	Nannong 87-17	SHRR	54.6	105.4	medium	Drought tolerance
#012	NJP493	SHRR	44.6	99.4	medium	Drought tolerance
#013	NJP556	NHRR	36.8	99.2	large	Drought tolerance
#014	Sidou 520	NHRR	36.5	96.6	large	Drought tolerance
#015	Xudou 15	NHRR	38.7	98.3	medium	Drought tolerance
#016	Xudou 18	NHRR	37.3	99.2	large	Drought tolerance
#017	NJP094	SHRR	53.4	106.8	large	Medium drought tolerance
#018	NJP101	NHRR	36.7	93.7	small	Medium drought tolerance
#019	NJP102	SHRR	40.0	97.2	small	Medium drought tolerance
#020	NJP108	NHRR	42.3	94.8	medium	Medium drought tolerance
#021	Jidou 7 Hao	Hebei	42.0	102.3	medium	Medium drought tolerance
#022	Heinong 26	Heilongjiang	43.8	101.7	medium	Medium drought tolerance
#023	Chudou 1	Anhui	41.3	98.7	large	Medium drought tolerance
#024	Liufeng	NHRR	37.3	93.3	large	Unknown
#025	NJP217	SHRR	50.2	105.6	large	Drought susceptibility
#026	L65-3366-C	USA	42.9	100.7	large	Medium drought tolerance
#027	Youchu 4	Beijing	39.5	98.2	medium	Medium drought tolerance
#028	Xinliuqing	Anhui	43.1	103.3	medium	Medium drought tolerance
#029	NJP279	SHRR	50.4	103.6	large	Medium drought tolerance
#030	Graham	USA	39.8	101.1	large	Medium drought tolerance
#031	NJP362	NHRR	37.5	94.5	large	Medium drought tolerance
#032	NJP367	NHRR	35.5	95.7	medium	Drought susceptibility
#033	NG5545	USA	43.6	99.1	small	Medium drought tolerance
#034	D76-1609	USA	46.1	102.6	large	Medium drought tolerance
#035	AGH	Anhui	38.9	98.1	small	Medium drought tolerance
#036	NJP405	NHRR	40.8	96.6	medium	Unknown
#037	NJP477	NHRR	42.5	102.0	small	Medium drought tolerance
#038	NJP542	SHRR	44.8	102.5	medium	Medium drought tolerance
#039	NJP580	SHRR	48.7	103.9	small	Medium drought

Code #	Name	Adaptive regions [#]	Days to anthesis (d) [€]	Days to maturity (d) [€]	Root system size [£]	Drought tolerance [$]
						tolerance
#040	Tongdou 7	SHRR	42.9	99.3	small	Medium drought tolerance
#041	S12C324	NHRR	38.2	103.0	large	Medium drought tolerance
#042	Xudou 13	NHRR	36.3	94.3	large	Medium drought tolerance
#043	NJP007	NHRR	38.0	97.6	medium	Drought tolerance
#044	NJP011	NHRR	37.8	96.0	medium	Drought tolerance
#045	Fendou 51	NHRR	37.3	99.3	medium	Drought susceptibility
#046	NJP051	SHRR	43.1	102.7	large	Drought susceptibility
#047	T173	Japan	40.5	101.3	small	Drought susceptibility
#048	NT-1	SHRR	38.9	101.4	medium	Unknown
#049	NT821058	NHRR	43.8	100.1	medium	Drought susceptibility
#050	NJP072	SHRR	42.8	99.7	medium	Drought tolerance
#051	NT821060	NHRR	45.8	101.4	large	Drought susceptibility
#052	Zhechun 3 Hao	Zhejiang	38.5	97.0	small	Drought susceptibility
#053	Hanchuan bayuebao	Hubei	53.2	102.6	large	High drought susceptibility
#054	NG6255	USA	41.2	102.4	small	Drought susceptibility
#055	Aijiaozao	Hubei	54.4	110.4	large	Drought susceptibility
#056	NT821096	NHRR	44.2	102.3	large	Drought susceptibility
#057	N7241	Anhui	39.8	96.7	small	Drought susceptibility
#058	B295	USA	40.5	100.8	large	Drought susceptibility
#059	HL05	USA	49.4	100.8	small	Drought susceptibility
#060	You 96-4	Hubei	44.1	96.9	small	Drought susceptibility
#061	NJP463	NHRR	46.8	108.2	small	Drought susceptibility
#062	NJP497	SHRR	36.2	96.8	small	Drought susceptibility
#063	SD007	NHRR	34.3	98.6	medium	Drought susceptibility
#064	LU-01	NHRR	40.7	97.4	medium	Drought susceptibility
#065	Y10043	NHRR	41.1	99.0	small	Drought susceptibility
#066	NJP567	NHRR	39.5	92.0	large	Drought susceptibility
#067	Suxian 21	SHRR	44.3	105.1	large	Drought susceptibility
#068	Tongdou 8	SHRR	48.9	103.7	small	Drought susceptibility
#069	Huaidou 7	NHRR	40.7	93.9	large	High drought susceptibility
#070	Xudou 14	NHRR	36.7	97.2	medium	Drought susceptibility
#071	Xudou 16	NHRR	37.6	96.1	small	Drought susceptibility
#072	NJP632	NHRR	37.8	97.6	large	Drought susceptibility
#073	TEPZ-G	SHRR	40.4	102.8	large	High drought susceptibility
#074	11QQ01	NHRR	40.6	96.1	large	Drought susceptibility
#075	NJP012	SHRR	45.0	101.3	small	Unknown
#076	NJP016	NHRR	35.9	96.3	large	Medium drought tolerance
#077	NJP020	SHRR	44.5	102.7	large	Drought susceptibility
#078	Hefeng 35	Heilongjiang	44.1	97.8	medium	Drought susceptibility
#079	NJP038	SHRR	42.9	100.3	small	High drought tolerance
#080	NJP040	SHRR	57.7	107.7	large	Drought tolerance
#081	NJP043	SHRR	44.3	102.1	large	Drought tolerance
#082	NJP044	NHRR	38.8	96.0	large	Medium drought tolerance
#083	NJP058	SHRR	33.6	98.8	large	Drought susceptibility
#084	NJP078	SHRR	36.7	97.8	large	Medium drought tolerance
#085	NJP089	SHRR	57.3	106.7	medium	Drought susceptibility
#086	NJP095	NHRR	37.3	97.3	small	Drought tolerance
#087	NJP100	SHRR	50.9	107.1	small	Medium drought tolerance
#088	NJP115	SHRR	44.2	102.8	small	Drought tolerance
#089	NJP121	SHRR	40.3	100.4	small	Drought tolerance
#090	Nanxiong huangdou	Guangdong	61.5	106.3	large	Drought tolerance
#091	NJP128	NHRR	42.3	100.6	large	High drought

Code #	Name	Adaptive regions [#]	Days to anthesis (d) [€]	Days to maturity (d) [€]	Root system size [£]	Drought tolerance [$]
						susceptibility
#092	NJP133	SHRR	40.8	102.3	small	Drought susceptibility
#093	NJP134	NHRR	39.3	95.1	large	Drought susceptibility
#094	NJP140	SHRR	43.1	104.0	large	Medium drought tolerance
#095	NJP149	SHRR	47.5	102.7	medium	Medium drought tolerance
#096	NJP161	NHRR	39.3	96.7	small	Unknown
#097	NJP180	NHRR	44.2	100.3	large	Drought tolerance
#098	IA2077	USA	38.2	95.3	small	High drought tolerance
#099	NJP191	SHRR	47.9	103.8	small	Drought tolerance
#100	NJP195	SHRR	45.5	101.7	large	Drought tolerance
#101	NJP196	SHRR	37.2	97.3	large	Drought susceptibility
#102	NJP212	SHRR	45.9	103.2	large	Medium drought tolerance
#103	NJP216	NHRR	47.4	100.4	small	Medium drought tolerance
#104	NJP221	NHRR	39.4	95.4	large	Drought susceptibility
#105	Taixing heidou	SHRR	34.7	99.4	medium	Drought tolerance
#106	NJP228	SHRR	54.4	104.2	large	High drought tolerance
#107	NJP233	SHRR	45.3	102.4	large	Drought tolerance
#108	Jilin 31	Jilin	35.4	93.0	small	Drought tolerance
#109	NJP250	NHRR	37.8	97.3	medium	Medium drought tolerance
#110	NJP251	NHRR	37.4	95.8	large	Medium drought tolerance
#111	NJP252	NHRR	41.4	96.6	large	Medium drought tolerance
#112	Vance	USA	42.4	102.3	small	Medium drought tolerance
#113	NJP259	SHRR	38.3	101.8	large	Drought susceptibility
#114	You 94-412	SHRR	38.2	96.6	small	Unknown
#115	NJP274	SHRR	38.6	99.4	medium	Medium drought tolerance
#116	NJP277	NHRR	38.6	98.1	small	Drought susceptibility
#117	NJP280	NHRR	36.8	96.6	large	Drought tolerance
#118	NJP289	NHRR	40.5	94.0	small	High drought susceptibility
#119	NJP302	SHRR	42.2	100.3	large	Medium drought tolerance
#120	NJP320	NHRR	37.9	95.7	medium	Drought susceptibility
#121	OT94-47-H	Canada	41.6	100.3	small	Drought susceptibility
#122	NJP329	NHRR	40.1	98.3	medium	Drought tolerance
#123	NJP340	SHRR	43.8	99.1	small	Medium drought tolerance
#124	83-19	Hubei	43.1	97.3	small	Drought tolerance
#125	NJP365	NHRR	38.5	91.7	medium	Drought susceptibility
#126	NJP374	SHRR	41.8	101.7	medium	Drought susceptibility
#127	NJP375	NHRR	38.7	97.4	medium	High drought susceptibility
#128	NJP383	SHRR	44.9	99.9	large	Drought susceptibility
#129	NJP393	SHRR	42.7	99.8	small	Medium drought tolerance
#130	NJP395	SHRR	37.3	97.2	small	Medium drought tolerance
#131	Chuxiu	NHRR	39.1	96.9	medium	Drought tolerance
#132	NJP421	SHRR	43.7	104.7	small	Drought susceptibility
#133	NJP436	NHRR	39.7	94.1	small	Medium drought tolerance
#134	NJP446	SHRR	45.6	98.9	small	High drought tolerance
#135	NJP449	SHRR	38.5	99.7	medium	Drought susceptibility
#136	NJP466	SHRR	46.3	103.8	large	Unknown
#137	NJP467	SHRR	39.8	96.6	small	Medium drought tolerance
#138	NJP471	SHRR	42.3	104.4	medium	Medium drought

Code #	Name	Adaptive regions #	Days to anthesis (d) €	Days to maturity (d) €	Root system size £	Drought tolerance $
						tolerance
#139	NJP474	SHRR	52.6	105.2	small	Medium drought tolerance
#140	NJP482	NHRR	46.8	105.2	small	Drought susceptibility
#141	NJP483	NHRR	45.9	105.4	medium	Drought susceptibility
#142	NJP487	NHRR	41.2	98.6	small	Drought susceptibility
#143	NJP498	SHRR	47.9	107.3	large	Drought tolerance
#144	NJP503	SHRR	40.5	100.3	small	Medium drought tolerance
#145	NJP505	SHRR	43.9	103.7	small	Drought susceptibility
#146	NJP507	SHRR	44.6	100.6	small	Drought tolerance
#147	NJP509	SHRR	46.4	101.2	small	Medium drought tolerance
#148	NJP519	NHRR	43.8	100.5	large	Drought tolerance
#149	NJP520	NHRR	42.3	99.9	large	Drought susceptibility
#150	NJP525	SHRR	45.3	100.2	medium	Medium drought tolerance
#151	NJP530	SHRR	48.9	105.5	large	Medium drought tolerance
#152	Nannong99-6	SHRR	44.7	101.2	large	Medium drought tolerance
#153	NJP553	NHRR	35.1	97.1	large	Drought susceptibility
#154	NJP558	NHRR	37.3	97.1	medium	Drought susceptibility
#155	Y-10-05	NHRR	38.6	98.2	medium	Drought tolerance
#156	NJP572	NHRR	44.8	105.1	medium	Drought tolerance
#157	NJP587	NHRR	41.3	99.0	small	Unknown
#158	Nannong 32	SHRR	52.5	109.3	small	Unknown
#159	Nannong 26	SHRR	50.0	105.8	large	Unknown
#160	Nannong 33	SHRR	53.7	104.5	large	Unknown
#161	Tongdou 2006	SHRR	49.0	106.3	medium	Unknown
#162	Dongxin 3	NHRR	36.3	96.0	large	Unknown
#163	Nannong 307-1	SHRR	40.4	90.6	small	Unknown
#164	Huaidou 9	NHRR	37.9	96.4	small	Unknown
#165	Huaidou 10	NHRR	43.3	94.8	medium	Unknown
#166	Huaidou 11	NHRR	40.7	97.3	small	Unknown
#167	Sidou 13	NHRR	36.9	94.0	large	Unknown
#168	Sudou 5	SHRR	39.8	96.4	small	Unknown
#169	Tongdou 6	SHRR	42.0	96.7	medium	Unknown
#170	Xudou 17	NHRR	37.2	96.6	medium	Unknown
#171	NJP635	SHRR	52.2	105.6	large	Drought susceptibility

Seeds were sourced from Soybean Research Institute, Nanjing Agricultural University, Nanjing, China.

Adaptive regions: NHHR, Northern Huaihe River region; SHHR, Southern Huaihe River region.

€, data of days to anthesis and days to maturity were provided by Prof Tuanjie Zhao according to an early study conducted in Nanjing, Jiangsu Province in 2018–2019.

£, root system size data were from this study (the semi-hydroponic system).

$, drought tolerance data were extracted from Wang et al, 2017 (Y.D. Wang, M.G. Xu, Y.J. Zhang, Y.Y. Weng, X.Y. Li, J.J. Kong, T.J. Zhao, X.H. He, Identification of drought-tolerance of soybean germplasms from Yangtze and Huaihe River Valleys at seedling stage, Soybean Sci 36 (2017) 669-678. https://doi.org/10.11861/j.issn.1000-9841.2017.05.0669).

Table S2. Soybean genotypes ranked in the top 15 (large values, indicated by ❋) or bottom 15 (small values, indicated by ★) among 171 genotypes from the phenotyping experiment for total root length (RL), with some also ranked in the top/bottom 15 for other traits.

Code #	Genotype name	RL	MRD	RM	SM	RL-upper	RL-lower
**Genotypes ranked in the top 15 for root length (RL)**							
**#055**	Aijiaozao	❋	❋	❋	❋	❋	❋
**#030**	Graham	❋		❋	❋	❋	❋
**#151**	NJP530	❋		❋	❋	❋	❋
**#001**	Kefeng 1	❋	❋	❋			❋
**#014**	Sidou 520	❋		❋	❋	❋	❋
**#091**	NJP128	❋	❋	❋			❋
**#110**	NJP251	❋		❋	❋		❋
**#051**	NT821060	❋					❋
**#081**	NJP043	❋		❋		❋	
**#006**	Nannong 86-4	❋		❋		❋	
**#077**	NJP020	❋		❋	❋	❋	
**#080**	NJP040	❋		❋	❋	❋	
**#067**	Suxian 21	❋		❋	❋	❋	
**#049**	t821058	❋					
**#042**	Xudou 13	❋				❋	
**Genotypes ranked in the bottom 15 for root length (RL)**							
**#002**	Nannong 88-31	★	★				
**#112**	vance	★		★	★	★	★
**#137**	NJP467	★				★	
**#108**	Jilin 31	★	★		★	★	★
**#052**	Zhechun 3 Hao	★		★		★	★
**#092**	NJP133	★	★	★	★	★	
**#133**	NJP436	★		★		★	
**#134**	NJP446	★	★	★	★	★	★
**#142**	NJP487	★	★		★	★	★
**#075**	NJP012	★	★	★	★	★	★
**#054**	NG6255	★	★	★	★	★	
**#040**	Tongdou 7 Hao	★		★	★	★	★
**#157**	NJP587	★		★	★	★	★
**#079**	NJP038	★	★	★	★	★	★
**#071**	Xudou 16	★	★	★	★	★	★

RL, total root length; MRD, maximal root depth; RM, root mass; SM, shoot mass; RL-upper, root length in 0–20 cm soil layer; RL-lower, root length below 20 cm soil depth.

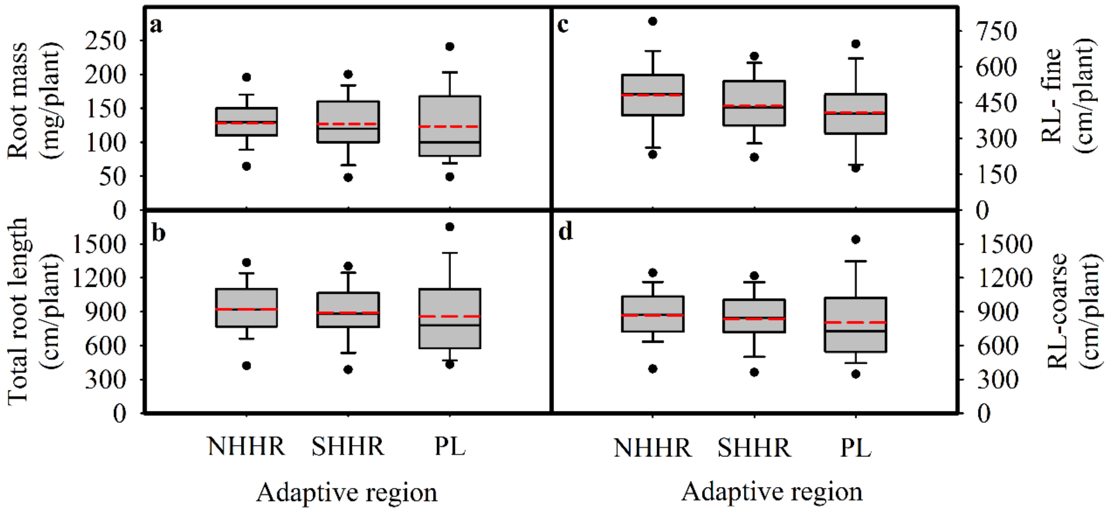

Fig. S1. Root mass (RM; a), total root length (RL; b), root length in diameter fine (RL-fine; c) and root length in diameter coarse (RL-coarse; d) for genotypes in three source categories NHHR (68 genotypes), SHHR (75 genotypes) and PL (28 genotypes) used in the phenotyping experiment. NHHR, Northern Huaihe River region; SHHR, Southern Huaihe River region; PL, Parental lines. The 171 genotypes were grown in a semi-hydroponic phenotyping platform 39 days after sowing. Significant differences are shown for three the source categories ($P \leq 0.05$). The whiskers, box and dot are determined by the 5th and 95th percentiles, 25th and 75th percentiles, and the 1st and 99th percentiles, respectively. The line and dashed inside the box marks are the median and mean, respectively.

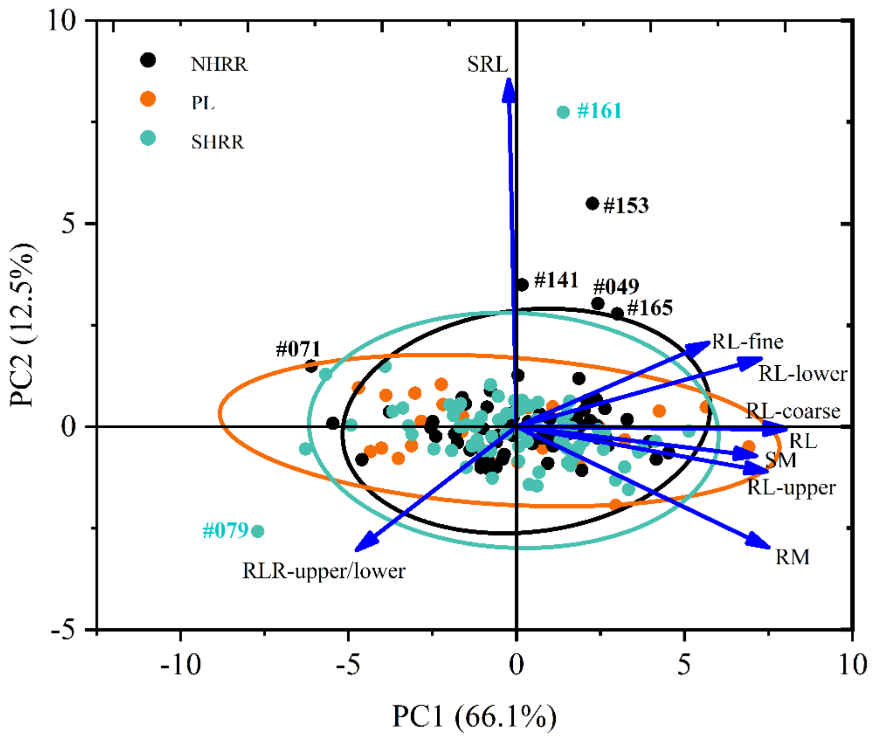

Fig. S2. Principal component analysis of nine selected traits with CVs ≥ 0.3 across 171 soybean genotypes grown in a semi-hydroponic phenotyping platform 39 days after sowing. The position of each trait is shown for PC1 vs. PC2 representing 78.6% of the total variability. The genotypes with extreme traits (outliers) are indicated by genotype number. NHHR, Northern Huaihe River region; SHHR, Southern Huaihe River region; PL, Parental lines.
